# Spatial Dynamics and Lifespan of Adult Cicadas After Fire and Logging: A Radiotracking Study

**DOI:** 10.1111/1749-4877.12970

**Published:** 2025-03-26

**Authors:** Carles Tobella, Marc Franch, Josep M. Bas, Lluís Brotons, Pere Pons

**Affiliations:** ^1^ Departament de Ciències Ambientals Universitat de Girona Girona Spain; ^2^ CICGE ‐ Centro de Investigação em Ciências Geo‐Espaciais Universidade de Porto Vila Nova de Gaia Portugal; ^3^ CTFC Solsona Spain; ^4^ CSIC Cerdanyola del Vallès Spain; ^5^ CREAF Cerdanyola del Vallès Spain

**Keywords:** fire refuges, *Lyristes plebejus*, radio‐tracking, salvage logging, wildfires

## Abstract

Recently burnt and logged habitats challenge the persistence of animal populations. Insects like cicadas, which survive belowground during fire and logging, are exposed to hostile conditions due to increased predation and limited resources when they emerge as adults. This study investigates the combined effects of wildfire and post‐fire salvage logging on the survival, spatial behavior, and habitat selection of the cicada *Lyristes plebejus* in Mediterranean pine forests. A total of 63 individuals were captured, tagged, and released across six plots in three disturbance contexts: burnt and logged, burnt and unlogged, and unburnt. Using radio telemetry, we tracked their movements and compared home range size and survival across these contexts. Results show that cicadas in burnt but unlogged areas were more mobile and tended to select areas of lower fire severity compared to those in burnt and logged areas. Salvage logging removed essential fire refuges, increasing exposure to predators. Although no significant differences in total distance covered were found, cicadas in burnt and logged areas displayed lower movement rates, indicating a reduced ability to explore and select suitable habitats. These findings highlight the importance of considering both wildfire and post‐fire management practices in conservation. Salvage logging exacerbates the negative effects of fire, emphasizing the need to preserve biological legacies and fire refuges to promote ecosystem resilience. The study suggests that careful forest management is crucial for protecting biodiversity, particularly for species like cicadas that depend on both above and below‐ground habitats.

## Graphical abstract

This study investigates the effects of wildfire and post‐fire salvage logging on the survival, spatial behavior, and habitat selection of *Lyristes plebejus* cicadas in Mediterranean pine forests. A total of 63 cicadas were captured, tagged with radio transmitters, and released in three disturbance contexts: burnt‐logged, burnt‐unlogged, and unburnt. Results indicate that cicadas in burnt‐unlogged areas were more mobile and preferred areas with lower fire severity. Survival was lowest in burnt‐logged areas, highlighting the need for fire refuges to enhance population resilience.

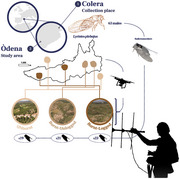

## 1 Introduction

Disturbances have the potential to significantly alter ecosystems, communities, and population structures (Turner 2010). Wildfires represent one such disruptive force. However, the characteristics of the fire regime, that is, fire extent, intensity, severity, seasonality, and frequency (Flannigan et al. 2009; Flannigan et al. 2013) are changing due to the anthropogenic modification of the Biosphere (Kelly et al. 2023). Within this framework, post‐fire forest management, often in the form of salvage logging, represents an additional disturbance (Lindenmayer et al. 2008; Hernández‐Hernández et al. 2017). While the impact of a single disturbance might be fairly well understood, multiple interacting disturbances can lead to unpredictable outcomes. These include more extensive damage, increased probabilities of disturbance recurrence, reduced ecosystem resilience, and potential regime shifts (Buma 2015). Postfire salvage logging often leads to interaction modifications rather than additive effects, disrupting the ecological roles of natural disturbances' biological legacies (Lindenmayer and Ough 2006; Noss et al. 2006). These disruptions can cause cascading effects and push ecosystems beyond recovery (Lindenmayer et al. 2008; Buma 2015).

Burnt forests have been logged more extensively over time due to changes in forestry practices across the globe (Müller et al. 2019; Leverkus et al. 2020). Stem‐only harvesting, which leaves low‐value timber remains on the ground, has recently given way to whole‐tree harvesting for bioenergy, using heavy machinery (Thorn et al. 2018; Leverkus et al. 2018). The environmental effects of salvage logging after wildfires are now an increasing concern (Pons and Rost 2017).

Recently burnt and logged habitats are a challenge for the persistence of animal populations. Insects that survive these disturbances, for example belowground, may sometimes show behavioral plasticity and manage to find adequate resources. However, the disturbed habitat could be unfavorable for their survival and reproduction due to increased predation and limited resources. Whether insects survive, die, or emigrate from burnt and logged areas depends on species‐specific traits and fire characteristics. Compared to the aboveground effects, the impacts of fire and logging on belowground forest biodiversity have received little attention (Swengel 2001; Crowther et al. 2014).

Pyrodiversity, that is, the variety of fire characteristics within an ecosystem, plays a crucial role in shaping biodiversity, particularly in fire‐prone landscapes. The pyrodiversity hypothesis suggests that a high diversity of fire events, differing in size, frequency, severity, and timing, promotes a corresponding increase in biological diversity by creating a mosaic of ecological niches that support diverse species (Martin and Sapsis 1992; Parr and Brockett 1999). Complex fire histories across space contribute to the spatial heterogeneity of communities, enhancing species turnover and overall biodiversity (Farnsworth et al. 2014; Burkle et al. 2015). Recent studies reinforce this hypothesis, showing that even modest increases in pyrodiversity can significantly boost the richness and interactions of plant‐pollinator communities (Ponisio et al. 2016). There is also evidence that pyrodiversity may be a positive predictor of overall bee and butterfly richness, diversity, and abundance (Ulyshen et al. 2022).

Cicadas (Cicadoidea) are abundant in diverse ecosystems worldwide, including forests, where they play key ecological roles (Marshall et al. 2018). Although they spend most of their life cycle underground as nymphs, their emergence as winged adults represents a highly mobile phase crucial for species dispersal and habitat selection. Cicada emergence shortly after a fire can provide valuable information about insect ability to disperse in severely transformed landscapes and select suitable habitat patches (Smith et al. 2006; Pons et al. 2023). Their capacity to disperse across fragmented or disturbed environments, such as burnt or post‐fire logged forests, makes them valuable for assessing the resilience of these ecosystems. By studying their movement patterns and habitat selection during this aerial phase, it is possible to evaluate the impact of forest disturbances and post‐disturbance management on population recovery. Despite their importance, the effects of fire and logging on cicadas during this critical dispersive phase remain poorly understood and warrant further investigation (Pons 2015).

In this study, we analyze whether the consecutive disturbances of wildfire and salvage logging (i.e., tree harvesting from forests affected by natural disturbances, typically to recover economic value), amplify adverse effects on survival, lifespan, movements, and home range of cicadas compared to wildfire alone and the absence of recent fire. To test this, we (1) quantified the spatial use and life‐history traits of cicadas across a gradient of disturbance severity and (2) identified suitable habitat patches, resulting from pyrodiversity, that are selected by cicadas. We hypothesize that combined wildfire and salvage logging will have the most adverse effects on cicadas, burnt‐unlogged areas will have intermediate effects, and cicadas will preferentially select habitat patches shaped by pyrodiversity. This research seeks to enhance our understanding of how disturbances affect cicadas and to inform strategies for effective habitat conservation.

## 2 Materials and Methods

### 2.1 Study Area and Sampling Design

**FIGURE 1:**
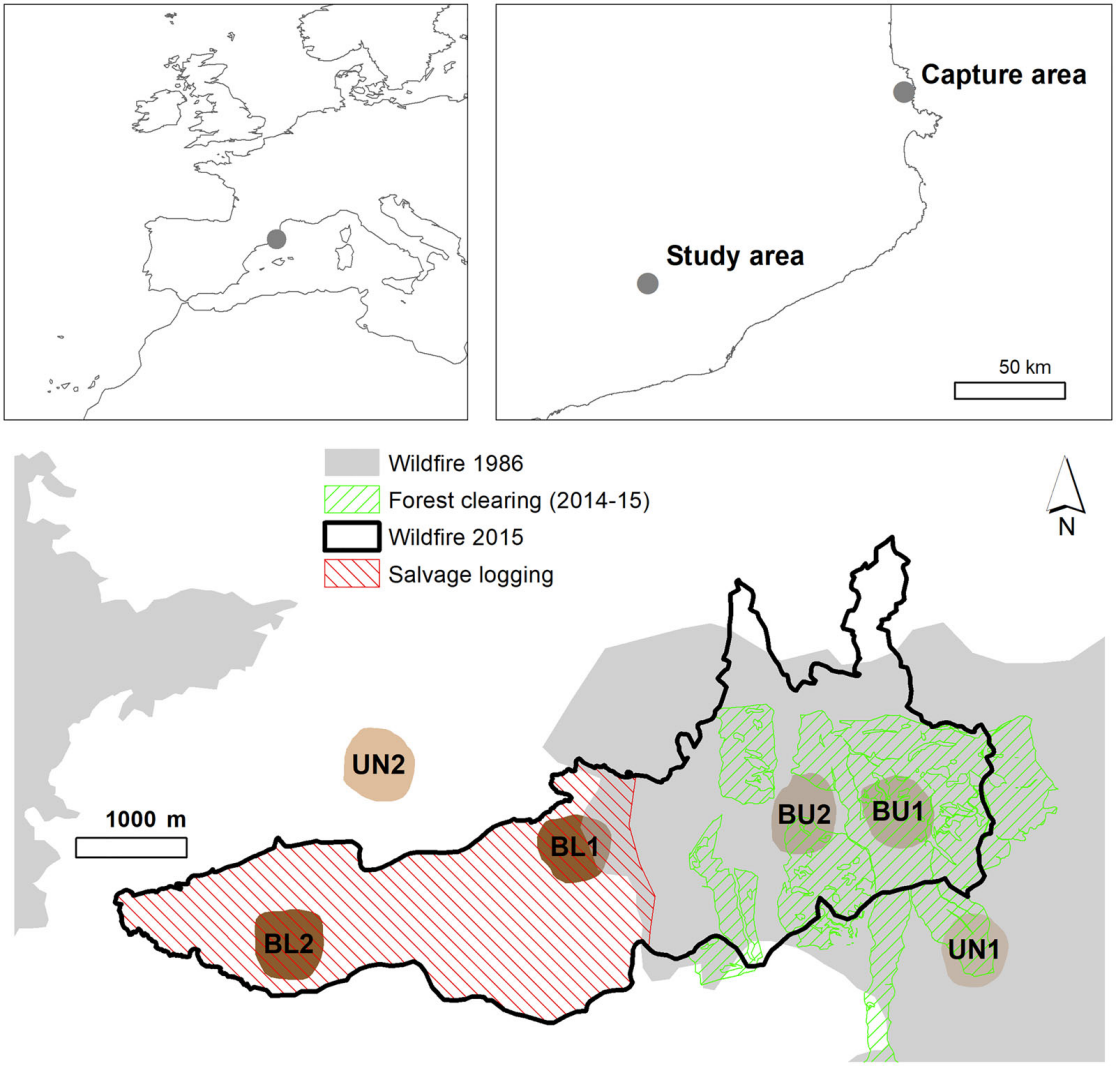
Location of the study region in Europe and study area in Catalonia (top maps) and map of the study plots and recent disturbances (bottom map). UN: unburnt, BU: burnt‐unlogged, and BL: burnt‐logged.

The study was conducted between June 27 and July 14, 2017, in an area burnt by wildfire in Òdena (Catalonia, NE Spain, 41.6316°N, 1.7187°E WGS84) and its surroundings. The Òdena wildfire burnt 1295 hectares between July 26 and 29, 2015. Historically, this area has experienced multiple impacts that have shaped the landscape. More intensely cultivated and grazed in the past by sheep and cattle, spontaneously afforested during the twentieth century, part of the study area was affected by a wildfire in 1986. The regenerating pine forest was cleared during 2014 and 2015, prior to the 2015 wildfire, to reduce tree density. Finally, the forest burned in 2015 was partially salvage‐logged (Table 1; Figure 1).

**TABLE 1:** Recent disturbances affecting each plot before the release of tagged cicadas.

Plot	Wildfire (1986)	Forest clearing (2014–2015)	Wildfire (2015)	Salvage logging (2015)
UN 1	1	1	0	0
UN 2	0	0	0	0
BU 1	1	1	1	0
BU 2	1	1	1	0
BL 1	0	0	1	1
BL 2	0	0	1	1

Abbreviations: UN: unburnt, BU: burnt‐unlogged, and BL: burnt‐logged plots. 1: Yes; 0: No.

Within this heterogeneous landscape, we selected six study plots representing three “disturbance contexts.” Two plots were located within the burnt area affected by salvage logging (BL), two plots within the burnt area not subjected to salvage logging (BU), and two plots in an unburnt area to serve as a control (UN) (Figures 1 and 4). Each treatment occupies a single continuous area, as shown on the map, which prevents the interspersion of plots between treatments BU and BL and may introduce a degree of spatial pseudoreplication. To ensure that the selected plots were representative of broader conditions, we based their selection on spatial data, including vegetation type, burn severity maps, and logging records. All plots were dominated by Aleppo pine (*Pinus halepensis* Mill, 1768) forests prior to the 2015 wildfire, ensuring consistency in habitat type across contexts. Additionally, the selected plots covered typical topographic and microhabitat features observed across the landscape for each disturbance context. This approach allowed us to compare cicada responses across a gradient of disturbance severity (BL, BU, and UN) thus minimizing confounding factors related to plot heterogeneity.

### 2.2 Focal Species and Individual Tracking

**FIGURE 2:**
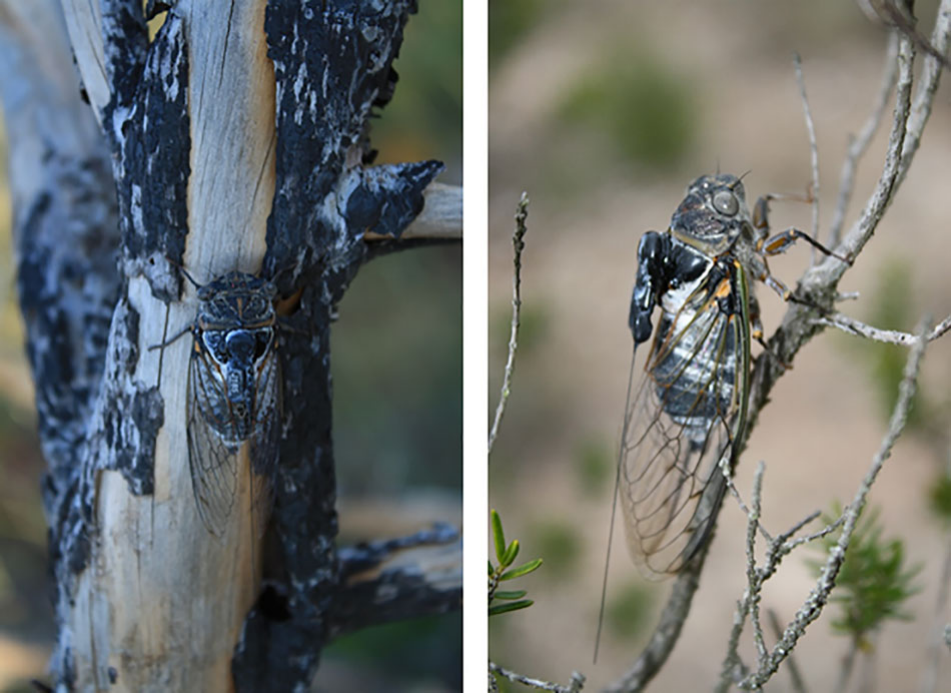
Tagged *Lyristes plebejus* with attached radiotransmitter on burnt and unburnt vegetation.

The study was conducted using males of *Lyristes plebejus* (Scopoli, 1763) (Hemiptera: Cicadidae). This species does not occur in the study area, although populations are known at approximate 9 km away, and it is the third commonest cicada species in Catalonia, the study region (Pons et al. 2021). *L. plebejus* is one of the largest cicadas in Europe. With an average weight of 1.84 g (*n* = 7 individuals), *L. plebejus* can fly while carrying a radio transmitter of 0.28 g, corresponding to an average of 16% of the insect's weight (Figure 2). To our knowledge, no other radiotracking studies have been conducted on cicadas, but similar methods have been applied to other insects, with transmitter‐to‐body‐mass ratios ranging from 2% to 100% (Kissling et al. 2014), and preliminary laboratory tests confirmed that the transmitter did not impair the flight capabilities. Cicadas were collected using a butterfly net in Colera (42.3969°N, 3.1366°E WGS84), in a healthy and dense population. To minimize disruption to the natural distribution of the species, we translocated only male *L. plebejus* individuals, preventing oviposition and the establishment of a new population in the study area. This approach also avoided genetic introgression, as no females were present locally, and ensured that the study did not disturb local ecological dynamics. These precautions allowed us to conduct the research responsibly while mitigating potential conservation concerns. Only fresh individuals were chosen, without worn wings and loss of pilosity, looking for recently emerged cicadas. Immediately after their capture, these individuals were translocated to our study area, situated 147 km to the SW, and distributed through the different treatment plots. Tagged individuals were released at the center of each plot, in locations separated at least 5 m from other released individuals to avoid interference.

We used radio telemetry, successfully applied to study the movement of individual insects such as carpenter bees (Pasquet et al. 2008), beetles (Hedin and Ranius 2002), Mormon crickets (Sword et al. 2005), and migrating dragonflies (Holland et al. 2006; Wikelski et al. 2006), to monitor cicada locations across time. A total of 26 Biotrack PicoPip Ag337 VHF transmitters (0.28 g and 20 days of lifespan) were used on our set of individuals, reusing them when individuals had died, across three time periods. This allowed the discontinuous monitoring of 63 cicadas in three batches, starting 7 days apart. The release dates of tagged individuals were June 27, 2017, July 4, 2017, and July 11, 2017. The radio transmitters were glued to the dorsal center of the mesonotum, after carefully removing its pilosity and applying a small drop of instant cyanoacrylate glue, followed by another small drop to the sides once the transmitter was attached. Two WMI TRX‐48S handheld receivers connected to a 3‐element folding yagi antenna (ATS F148‐3FB) were used to track the tagged cicadas, detecting the individual and recording its position, substrate, altitude, and behavior at least once a day. This procedure was repeated until the transmitters stopped transmitting or the individual was found dead. All this information was superimposed on detailed photocartography projected onto a Samsung Galaxy Tab S2 tablet.

### 2.3 Mapping of Fire Severity and Vegetation Recovery

High spatial resolution imagery was acquired using an unmanned aerial system (UAS; Phantom 3, DJI) to map unburnt forest patches and other potential suitable habitats within the study area. The UAS is a lightweight quadcopter (1.28 kg) equipped with an RGB digital camera featuring an f/2.8 lens, a 94° field of view, and a resolution of 12 megapixels. The camera captured HD video at up to 30 frames per second, producing images with a pixel resolution of 5 cm.

Two key variables were calculated to assess fire impact and vegetation recovery. Both variables were derived from Sentinel‐2 satellite imagery. The normalized burn ratio (NBR) was computed using near‐infrared (NIR; Band 8) and shortwave infrared (SWIR; Band 12) bands, following the equation:

$$\mathrm{NBR}\;=\frac{\mathrm{NIR}-\mathrm{SWIR}}{\mathrm{NIR}+\mathrm{SWIR}}$$

‐ Fire severity (dNBR Fire): The difference in the NBR before and immediately after the fire and was calculated as:
$$\mathrm{dNBR}\;\mathrm{Fire}\;=\left(\mathrm{NBRpre}-\mathrm{NBRpost}\right)\times 1000$$
‐ Vegetation recovery (dNBR Post): The difference in NBR values measured 23 months post‐fire, compared to the pre‐sampling period.

Pre‐fire imagery was acquired on July 16, 2015, 10 days before the wildfire's onset. Subsequently, post‐fire imagery was obtained on August 2, 2015, 4 days after the fire was extinguished. To calculate the dNBR post variable, pre‐sampling imagery from August 2, 2015, was compared with post‐sampling imagery captured on July 30, 2017. This integration of high‐resolution UAS data with satellite‐derived NBR metrics provided a robust method for quantifying fire impact and subsequent vegetation dynamics.

### 2.4 Data Analysis

We measured flight distances and home range sizes for each individual by calculating the minimum convex polygon (MCP), kernel 50, and kernel 95, where kernel 50 refers to the core area representing 50% of an animal's utilization distribution and kernel 95 encompasses 95% of the utilization distribution, indicating the broader area of activity. All were used to compare the spatial dynamics of cicadas between treatments.

To evaluate the cicada survival (*S*), we used a known‐fate model in the program MARK (White and Burnham 1999) with the logit‐link function. The model includes three assumptions: (1) attaching a transmitter to an individual does not affect its fate, (2) the fates of radio‐marked individuals are independent, and (3) tagging with transmitters is not related to mortality (White and Burnham 1999).

We used daily time periods for data recording of radio‐tracked cicadas. The possible individual multi‐surveys per day were converted to daily encounter histories for each monitored cicada (White and Burnham 1999). We censored individuals if we were unable to track them in a given sampling occasion. This sampling occasion must be between the release (*t* = 0) and the confirmed death of the individual (*t* = *n*) (Windels and Belant 2016).

We compared five a priori models: (1) constant survival *S*(.), (2) survival varying with time *S*(*t*), (3) survival as a function of disturbance context *S*(*g*), (4) survival as a function of disturbance context and time interaction *S*(g*t), and (5) survival varying with the additive effect of disturbance context and time *S*(*g* + *t*). Model selection was performed using Akaike's information criterion corrected for small sample size (AICc) (Akaike 1973; White and Burnham 1999), and models differing by ≤2 ΔAICc were considered as potential alternatives (Anderson and Burnham 2002).

## 3 Results

A total of 63 *L. plebejus* individuals, distributed across three batches, were collected, radio‐tagged, and tracked within six plots, across three disturbance contexts (Table 2).

**TABLE 2:** Number of individuals released per disturbance context, plot, and batch.

Disturbance context		Batch 1	Batch 2	Batch 3	All
UN	UN 1	4	3	2	9
	UN 2	4	3	3	10
	Sum	8	6	5	19
BU	BU 1	5	3	4	12
	BU 2	4	2	3	9
	Sum	9	5	7	21
BL	BL 1	5	4	3	12
	BL 2	6	3	2	11
	Sum	11	7	5	23
	All	28	18	17	**63**

Abbreviations: UN: unburnt, BU: burnt‐unlogged, and BL: burnt‐logged plots.

We conducted radio‐tracking of tagged cicadas over 18 days, resulting in a total of 286 locations: 87 for UN, 113 for BU, and 86 for BL plots. The number of locations per tracked individual was 4.54 ± 2.49 (mean ± SD), ranging from 2 to 13 (Table 3).

**TABLE 3:** Number and basic statistics of individual locations obtained for three disturbance contexts: UN: unburnt, BU: burnt‐unlogged, and BL: burnt‐logged plots.

	*N* of locations	Mean	SD	Min	Max
UN	87	4.59	1.64	2	8
BU	113	5.38	3.43	2	13
BL	86	3.74	1.81	2	8
All	**286**	**4.54**	**2.49**	**2**	**13**

### 3.1 Movements and Home Ranges of Tagged Cicadas

**FIGURE 3:**
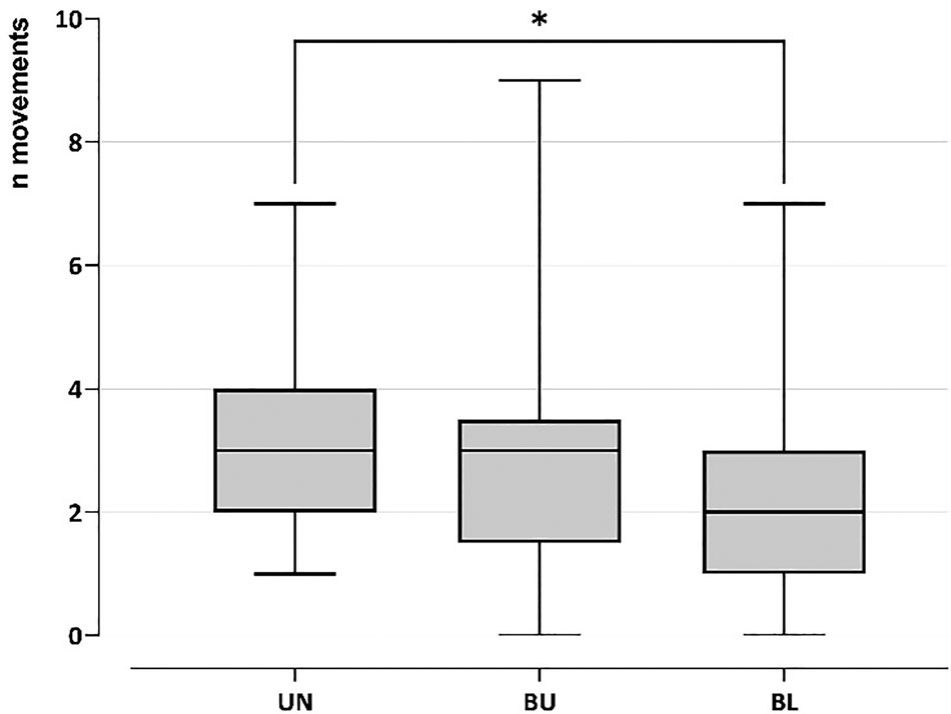
Number of movements of individual cicadas in the three treatments: UN: unburnt, BU: burnt‐unlogged, and BL: burnt‐logged plots. Centre line: median; box: first quartile (Q1) to third quartile (Q3); whiskers: min. to max.

**FIGURE 4:**
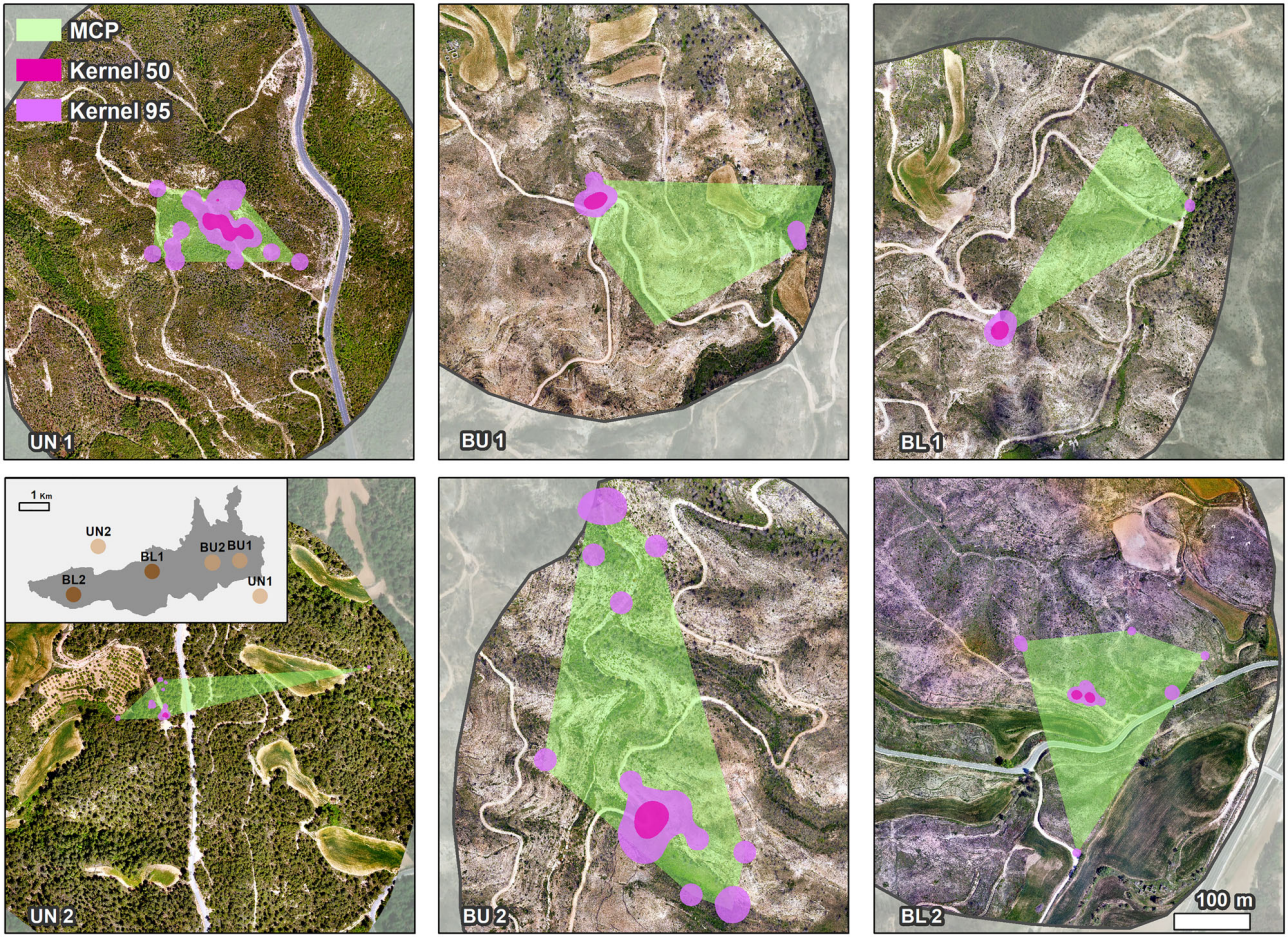
Graphical representation of the MCP, kernel 50, and kernel 95 of the total number of individuals marked per plot, plotted on the images obtained by UAS. UN: unburnt, BU: burnt‐unlogged, and BL: burnt‐logged plots.

The mean number of movements per individual was 2.82 ± 1.96 (mean ± SD), ranging from 0 to 9 (Table 4). Significant differences were detected between the three treatments (Kruskal–Wallis test: *H*_2,63_ = 6.4053, *p* = 0.0394), with pairwise comparisons showing differences only between unburnt and burnt‐logged areas (*p* = 0.0432). Overall, the mean number of movements was higher in unburnt than in burnt zones (Table 4; Figure 3 and 4).

**TABLE 4:** Number of movements and distances obtained for three disturbance contexts: UN: unburnt, BU: burnt‐unlogged, and BL: burnt‐logged plots.

					Distance (m)							
	Num. of movements				Per movement				Sum of distances			
	*n*	Mean	SD	Max	*n*	Mean	SD	Max	*n*	Mean	SD	Max
UN	19	3.32	1.63	7	63	19.67	44.62	337	19	65.21	95.97	404
BU	21	3.05	2.25	9	64	49.31	80.99	414	21	157.78	250.51	889
BL	23	2.21	1.91	7	52	26.63	66.74	312	23	69.24	106.46	332
ALL	**63**	**2.82**	**1.96**	**9**	**179**	**32.66**	**66.79**	**414**	**63**	**91.73**	**166.46**	**889**

No significant differences were detected in the average distance covered per movement (Kruskal–Wallis (K‐W) test: *H*_2,__63_ = 4.0508, *p* = 0.1319), in the sum of distances per individual (K‐W test: *H*_2,63_ = 4.9118, *p* = 0.0858), or in the maximum distance per movement per individual (K‐W test: *H*_2,63_ = 3.3609, *p* = 0.1863) between disturbance contexts.

The mean size of the MCPs for all released individuals was 1795 ± 5184 m^2^ (mean ± SD), 1998 ± 4212 m^2^ for 50% kernel density estimate (50% KDE), and 7941 ± 66650 m^2^ for 95% kernel density estimate (95% KDE) (Table 5). No significant differences were found between treatments.

**TABLE 5:** Summary table of MCP, 50% KDE (kernel density estimate), and 95% KDE areas obtained for the three disturbance contexts: UN: unburnt, BU: burnt‐unlogged, and BL: burnt‐logged plots.

	MCP (m^2^)				50% KDE (m^2^)				95% KDE (m^2^)			
	*n*	Mean	SD	Max	*n*	Mean	SD	Max	*n*	Mean	SD	Max
UN	16	307	488	1687	16	654	1833	7392	18	3198	9070	37663
BU	16	4203	8223	28420	19	2842	5338	17769	20	10619	20088	66649
BL	13	662	1276	3886	15	2361	4323	11619	17	8412	17234	51685
All	**45**	**1795**	**5184**	**28420**	**50**	**1998**	**4212**	**17769**	**55**	**7508**	**16307**	**66649**

### 3.2 Cicadas Spatial Use Across the Fire Severity Gradient

Fire severity was heterogeneous in BU plots because dNBR fire showed significant differences between real locations and randomly generated locations (Null BU) (Mann–Whitney's (M‐W's) *U* Test: *Z* = −0.3268, *n*BU = 64, *n*Null BU = 1104, *p* = 0.0199). Similarly, significant differences in vegetation growth were found when comparing dNBR post between real and randomly generated locations (Null BU) (M‐W *U* test: *Z* = 3.1794, *n*BU = 64, *n*Null BU = 1104, *p* = 0.0014). For BL plots, significant differences were observed only for dNBR Fire (M‐W *U* test: *Z* = 2.7431, *n*BL = 52, *n*Null BL = 1074, *p* = 0.0061).

**FIGURE 5:**
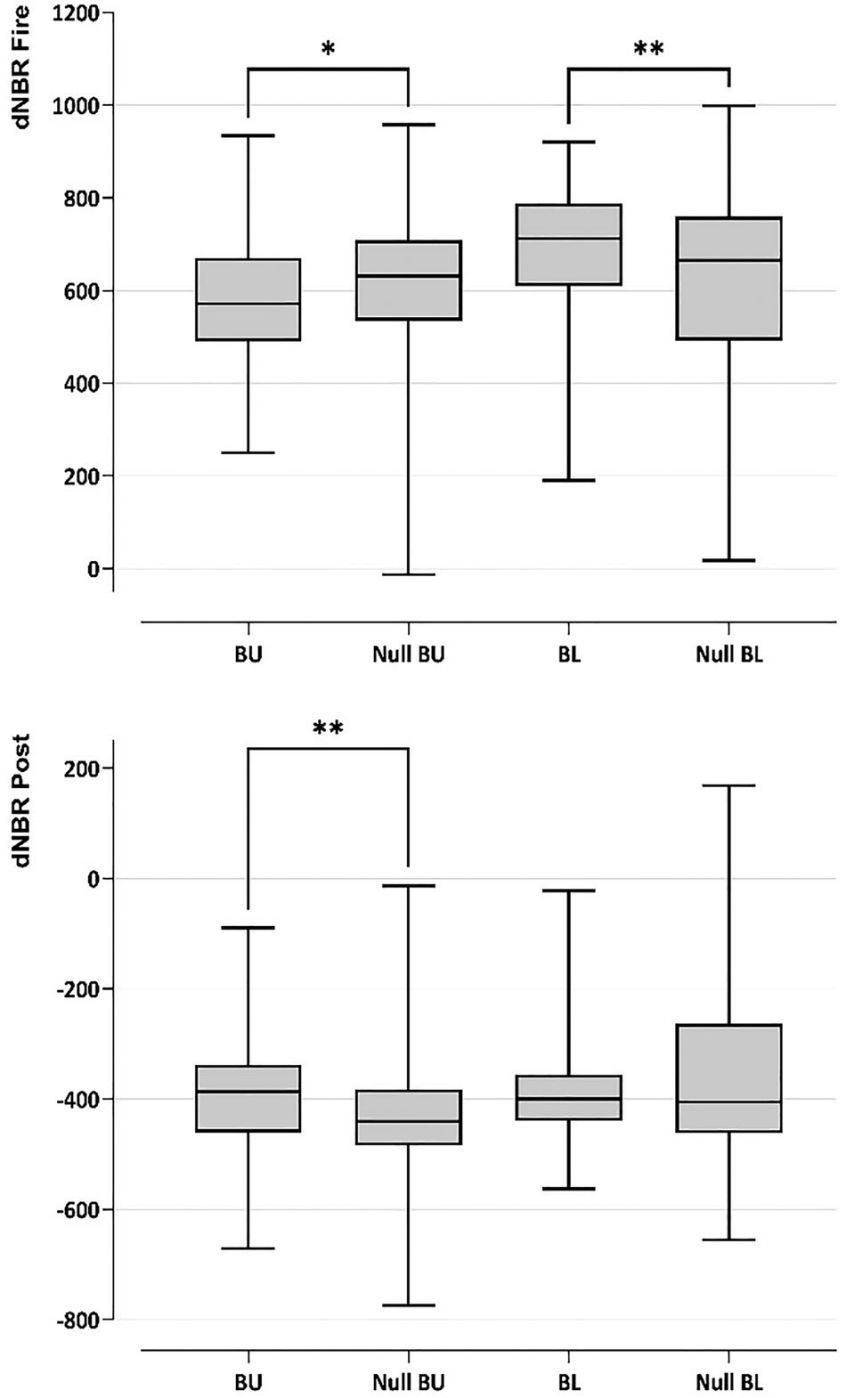
Fire severity (dNBR Fire) and vegetation growth (dNBR Post) of the tagged cicadas real locations (burnt‐unlogged, BU, and burnt‐logged plots, BL) compared to those randomly generated from the null models (Null BU and Null BL). Centre line: median; box: first quartile (Q1) to third quartile (Q3); whiskers: min. to max.

Tracked cicadas were predominantly found in areas of lower fire severity (lower dNBR Fire) in the BU treatment, whereas they were predominantly found in areas of higher fire severity in the BL treatment (Figure 5). Tracked cicadas were also found at sites with low vegetation growth (lower dNBR Post) during the post‐fire period in the BU treatment (Figure 5). The way in which released cicadas move and establish their home ranges may suggest a deliberate pattern.

**FIGURE 6:**
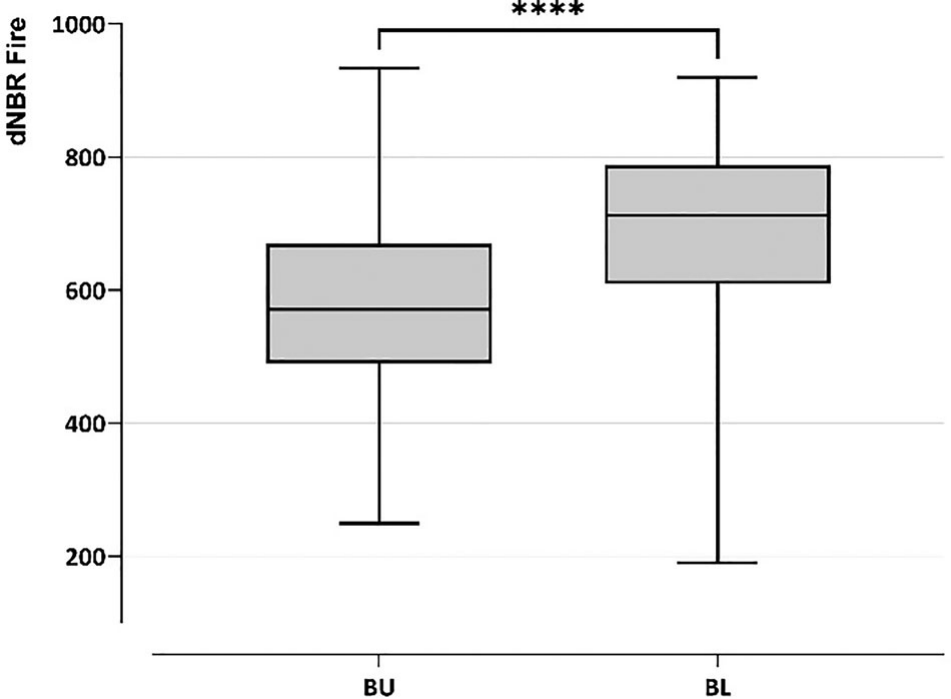
Differences in fire severity between burnt disturbance contexts. BU: burnt‐unlogged and BL: burnt‐logged plots. Centre line: median; box: first quartile (Q1) to third quartile (Q3); whiskers: min. to max. Significant differences are shown with **** (*p* > 0.0001).

The fire severity index (dNBR Fire) also showed significant differences between the BU and BL disturbance contexts (M‐W's *U* Test: *Z* = 5.0268, *n*BU = 64, *n*BL = 52, *p* < 0.0001) (Figure 6). However, no significant differences were found between the disturbance contexts for post‐fire plant growth (dNBR post).

**FIGURE 7:**
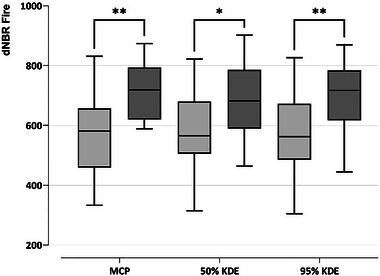
Fire severity (dNBR Fire) in cicada home ranges in burnt‐unlogged (BU, light gray) versus burnt‐logged (BL, dark grey) plots. Home ranges are measured as MCP, kernel 50, and kernel 95. Significant differences are shown with * (*p* > 0.05) and ** (*p* < 0.01).

Fire severity (measured by dNBR Fire) was significantly different between home ranges located in BU and BL plots. Specifically, home ranges (MCP, 50% KDE, and 95% KDE) in BU plots were located in areas of lower fire severity compared to BL plots (Figure 7). Conversely, no significant differences were found between home ranges in BU and BL plots for dNBR Post (Table 6).

**TABLE 6:** Summary of dNBR statistics including sample size (*n*), mean, standard deviation (SD), and maximum (Max) values (dNBR is a dimensionless index) for Fire MCP, Fire 50% KDE, Fire 95% KDE, and corresponding post‐fire metrics in BU: burnt‐unlogged and BL: burnt‐logged plots.

	BU				BL			
	*N*	Mean	SD	Max	*n*	Mean	SD	Max
dNBR Fire MCP	16	574.83	145.73	832	11	712.04	94.04	874
dNBR Fire 50% KDE	19	581.69	134.38	822	15	691.62	126.90	902
dNBR Fire 95% KDE	20	567.96	135.62	826	17	700.58	112.39	869
dNBR Post MCP	16	−411.41	95.58	−285	11	−399.73	54.29	−334
dNBR Post 50% KDE	19	−415.85	83.50	−289	15	−402.45	66.98	−226
dNBR Post 95% KDE	20	−406.70	83.48	−288	17	−390.12	57.71	−224

Movements and home ranges appeared to be directed toward or located in areas of low fire severity. When considering whether individuals moved into an unburnt vegetation patch or not, significant differences were found between BU and BL (*χ* ^2^_1, 55_ = 4.1251, *p* = 0.0422), with BU plots showing a high proportion of movements into unburnt vegetation patches (Table 7). However, the number of movements ending in an unburnt vegetation patch was not significantly different between BU and BL plots. Both the total area and the average size of unburned patches were larger in BU than in BL. All individuals that moved to unburnt patches selected these fire refuges until they died or stopped signaling.

**TABLE 7:** Description of the unburnt vegetation patches in each burnt plot and contacts of tagged cicadas in those patches.

Plot	Number of unburnt patches	Total area of patches (m^2^)	Mean area of patches (m^2^)	SD area of patches (m^2^)	Number of patches used by cicadas	Number of cicada contacts	Mean distance of contacts to the release site (m)
BL1	6	8531	1421	218	1	2	294
BL2	11	3731	339	2591	1	1	212
BU1	5	20773	4154	4897	1	6	270
BU2	1	10540	10540	—	1	6	369

Abbreviations: BU: burnt‐unlogged, BL: burnt‐logged, SD: standard deviation.

### 3.3 Lifespan of Tagged Cicadas

**FIGURE 8:**
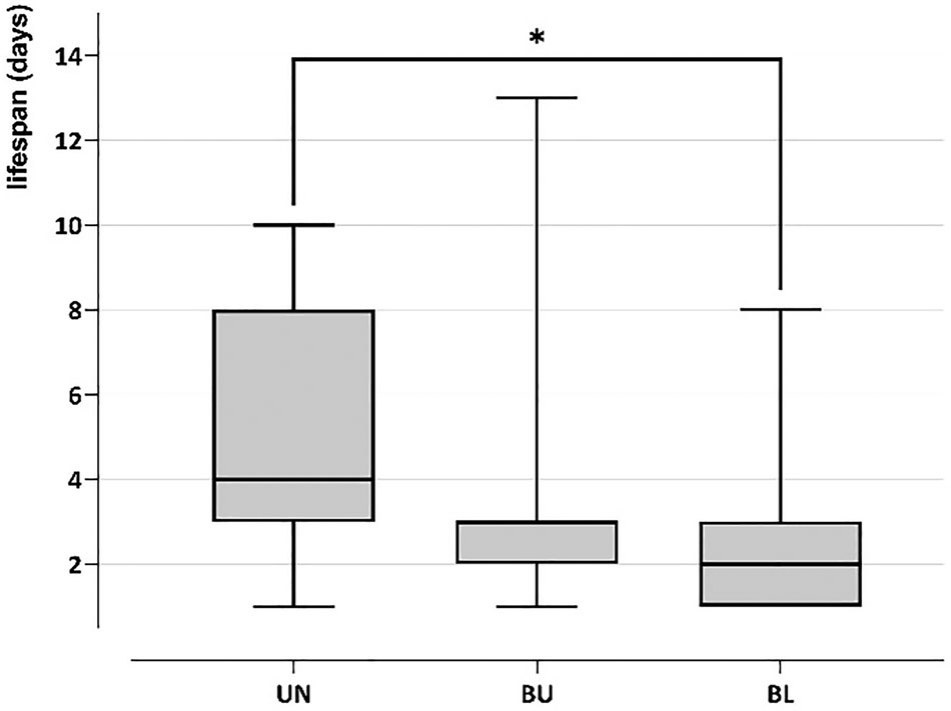
Lifespan, in days since release, of cicadas in the three disturbance contexts: UN: unburnt, BU: burnt‐unlogged, and BL: burnt‐logged plots.

Radiotracked cicadas had a mean individual longevity of 3.92 ± 3.09 days since their release (*n* = 63) (Figure 8). Longevity was significantly different between the three treatments (Kruskal–Wallis test: *H*_2,63_ = 6.4662, *p* = 0.0394). Pairwise comparisons revealed differences only between unburnt and burnt‐logged plots (*p* = 0.0394), with a higher mean lifespan in unburnt than in burnt areas.

Of the five candidate models selected to examine the influence of the variables considered on survival, the interaction model including treatments and time was the most parsimonious. The remaining four models were not considered to be competitive (> 2.00 ΔAICc).

**FIGURE 9:**
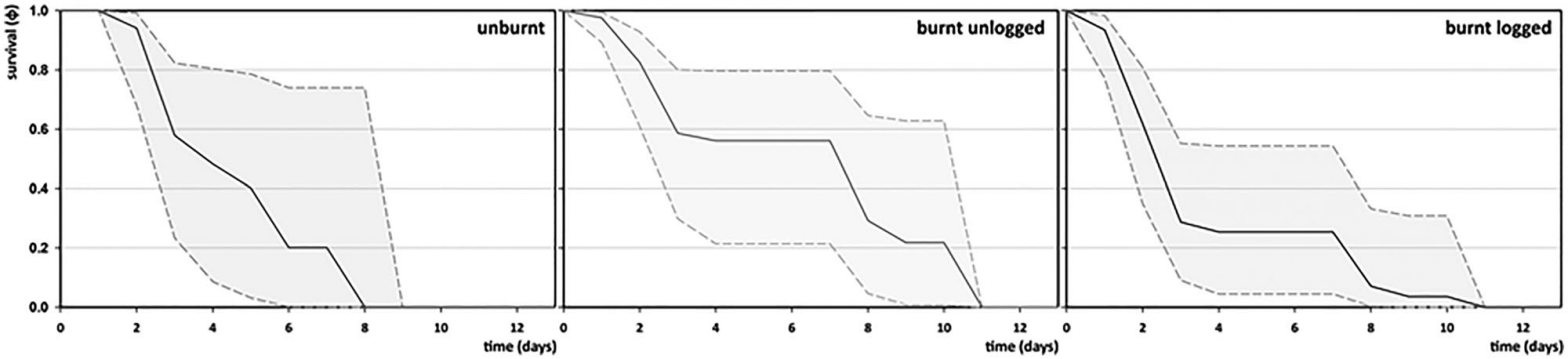
Survival rates (ɸ) of released cicadas in the three disturbance contexts across time.

For all individuals, survival during the 16‐day study period was 0.1521^−45^ and individual survival for disturbance contexts separately was even lower (Table 7). Among the three disturbance contexts, the tagged cicadas at BU plots were characterized by the highest daily survival rate (ɸ = 84.90%) and lower population extinction rates (ɸ_50_ = 6.15 days; ɸ_10_ = 12.05 days and ɸ_0_ = 13.52 days) (Figure 9; Table 8).

**TABLE 8:** Survival rate of tagged cicadas across the study period (16 days) (ɸ) population survival, and population extinction rate in days: (ɸ_50_) to reach 50% of the initial sample, (ɸ_10_) to reach 10% of the initial sample, and (ɸ_0_) sample extinction in UN: unburnt, BU: burnt‐unlogged, and BL: burnt‐logged plots.

	*n*	Survival ɸ					
		Survival (ɸ)		% population survival (ɸ) per day	Pop. extinction rates (days)		
		Estimate	SD		ɸ_50_	ɸ_10_	ɸ_0_
UN	19	0.22^−99^	—	75.09	4.75	10.73	12.22
BU	21	0.18^−94^	—	84.90	6.15	12.05	13.52
BL	23	0.79^−98^	—	61.50	3.12	10.50	12.35
ALL	**63**	**0.15^−45^**	**0.19^−41^**	**71.75**	**4.48**	**10.88**	**12.48**

During the study, we detected a high mortality rate associated with predation, reaching 67% (*n* = 33) of the released specimens. Predation rates varied between disturbance contexts, with almost half of the specimens released in control areas (UN) being predated (47%, *n* = 7). Predation rates were higher in burned disturbance contexts, reaching 81% (*n* = 13) in BU and 72% (*n* = 13) in BL plots, although these differences were not statistically significant.

## 4 Discussion

The movements and home ranges of released cicadas appear to be influenced by the effects of wildfire (a single disturbance) and subsequent salvage logging (representing two interacting disturbances). Initially, we hypothesized that our treatments would differentially affect cicada populations, with the greatest effect observed in the burned and logged plots, followed by the burned and unlogged plots, and the least effect in the unburned control. However, part of our results did not follow this pattern, as we discuss below. *L. plebejus* occupies tall shrublands, forests, and gardens, preferring habitats with variable horizontal cover and requiring some vertical structure (Puissant 2006). Therefore, we would expect movements to be short and limited in a suitable environment that meets the requirements for feeding and mimicry to avoid predation. This expectation aligns with our results in unburnt forest plots, where cicadas exhibited lower movements and home range size than the other two disturbance contexts. In contrast, they showed an unexpected response in burnt areas. The burnt and logged plots showed lesser movements and home ranges (Kernels and MCP) on average than burnt and unlogged plots, while burnt and unlogged plots recorded the longest movements and larger home ranges, most of them directed to unburnt patches.

As cicada survival is lower in plots disturbed twice, the time available for movements and the possibility of a large home range would be lower. Reduced movement ranges and survival rates in logged areas may stem from resource scarcity, habitat changes, or increased predation risks. Predator density often declines post‐fire, requiring time for recolonization (Mutz et al. 2017; Puig‐Gironès and Pons 2020), while prey concealment relies on mimicry and structural refuges like canopy cover, which are lost after wildfires, disrupting predator–prey dynamics. Moreover, being present in a degraded habitat could affect the physical condition of the cicadas and cause reduced movement. It is important to note that in some cases the area of the MCPs was smaller than that of the kernels because of a low locations sample size (Figure S1).

There is no information on the dispersal or home range size of *L. plebejus*, although it is known that females are attracted by male songs and fly toward singing males (Boulard 1988). Thus, by studying only males, we avoided considering these chorus‐seeking movements and focused solely on dispersal movements to select a suitable habitat. In the case of *Cicada orni* Linnaeus, 1758, a syntopic species with *L. plebejus*, most studied males cover less than 100 m and never exceed 150 m in olive tree crops (Simoes and Quartau 2007). Although American periodical cicadas, including males and females, have been observed moving up to 300 m (Williams and Simon 1995), long‐distance dispersal appears to be rare in cicadas (Maier 1982; Arensburger et al. 2004). We found that in undisturbed environments, *L. plebejus* moved at a median rate similar to *C. orni*, but occasionally moved over 400 m. In disturbed environments, these distances would be greater, reaching 890 m in our study. It is important to note that *L. plebejus* is approximately twice the size of *C. orni* and has good flight capacity. Insect responses to managed and natural fires align with patterns observed in other species. Studies such as Shapiro (1965), Williams (1988), and Swengel and Swengel (1997) highlight that fires can create favorable habitats for butterflies and other insects, provided there are unaffected areas for potential recolonization (Gervais and Shapiro 1999). However, our study uniquely evaluates the combined effect of wildfires and salvage logging, offering new insights into how these practices together influence cicada populations, a dimension largely unexplored in previous research.

After several years of living underground, nymphs emerge from the end of May to August in the study region (Pons et al. 2021). Once having emerged, adults live only a few days to mate and lay eggs. Males of *C. orni* can survive for about 2 weeks, but the majority likely live less than a week (Simoes and Quartau 2007). For *L. plebejus*, we found similar values, with a mean lifespan of around 5 days in the undisturbed plots, 4 days in the burnt‐unlogged, 3 days in the burnt‐logged plots, and up to a maximum of 13 days for those individuals that managed to find unburnt patches. Notably, the median lifespan was only 2.86 days in burnt and logged plots. The low survival can in part result from poorer habitat conditions, offering less resources that would result in a decreased health condition. Examination of dead cicadas showed that mortality was mostly associated with predation, probably due to the lack of refuges.

Movements and habitat use of our cicadas tended toward areas of low fire severity. These areas, often unburnt patches within the fire perimeter, can be considered fire refuges (Krawchuk et al. 2020). Interestingly, a greater number of individuals moved to unburnt areas in burnt and unlogged patches, where these areas are larger, compared to burnt and logged patches which have more unburnt patches in total but are smaller, and less attractive. This pattern could be the result of salvage logging which often does not respect these valuable refuges (Pons et al. 2020), which have a similar habitat structure to that of unburnt control forests. It is worth noting that the study area has gentle slopes, which allowed the cicadas to detect the unburnt patches with relative ease, as they were visible and easily recognizable from the release areas. Although the spatial configuration of the treatments may involve pseudoreplication due to the continuous nature of the treatment areas, we believe this does not significantly affect the results of the study. Specifically, differences in habitat structure between burned unlogged and burned logged plots at the time of the study were primarily driven by forestry interventions rather than spatially related factors, ensuring the robustness of the inter‐treatment comparison.

At least 106 Western Palearctic breeding bird species have been found to feed on cicadas, representing 16% of the bird species occurring in this region (Pons 2020). Juvenile survival of species that select cicadas as their primary prey may therefore increase in response to locally high cicada densities (Patterson et al. 1991). In the Mediterranean Basin, cicadas are a preferred food for some bird species, such as jays, that concentrate their movements in the pine forests where cicadas are abundant (Patterson and Cavallini 1996; Patterson et al. 1997; Rolando 1998). In our study area, unburnt forest patches were found to attract the tracked cicadas, as well as other species such as *C. orni* (personal observation). Keeping these unburnt patches unlogged can be considered crucial for post‐fire management, as it would support the persistence of cicada populations and, consequently, the species that rely on them as prey or host. Furthermore, this study raises important questions regarding the characteristics of fire refuges used by cicadas, particularly the minimum size and spatial distribution required for them to function as optimal zones for post‐fire recovery. Determining these parameters will necessitate further research, yet a coarse estimation of the unburnt patch minimum size (from 315 to 11169 m^2^) and maximum separation distance (from 212 to 417 m) is possible by considering the movement patterns to unburnt patches in this study. This highlights the need for integrative management strategies that prioritize the preservation of unburnt patches, given their critical role in maintaining food resources for higher trophic levels. Therefore, ensuring the protection and strategic placement of these refuges could significantly enhance conservation efforts and post‐fire ecosystem resilience.

## Supporting information




**Figure S1** Example explaining why in some cases the MCPs have a lower value than K50 and K95. This usually occurs when calculations are generated from a sample with few points.
