# Supplementary material for: Spatial Dynamics and Lifespan of Adult Cicadas After Fire and Logging: A Radiotracking Study
Source: Integr Zool. 2025 Mar 26;21(2):359–70. doi: 10.1111/1749-4877.12970 (PMC12971630; doi:10.1111/1749-4877.12970)
Supplement: Supplementary file 1 — Figure S1 Example explaining why in some cases the MCPs have a lower value than K50 and K95. This usually occurs when calculations are generated from a sample with few points. [file INZ2-21-359-s001.docx]

**SUPPLEMENTARY MATERIALS**


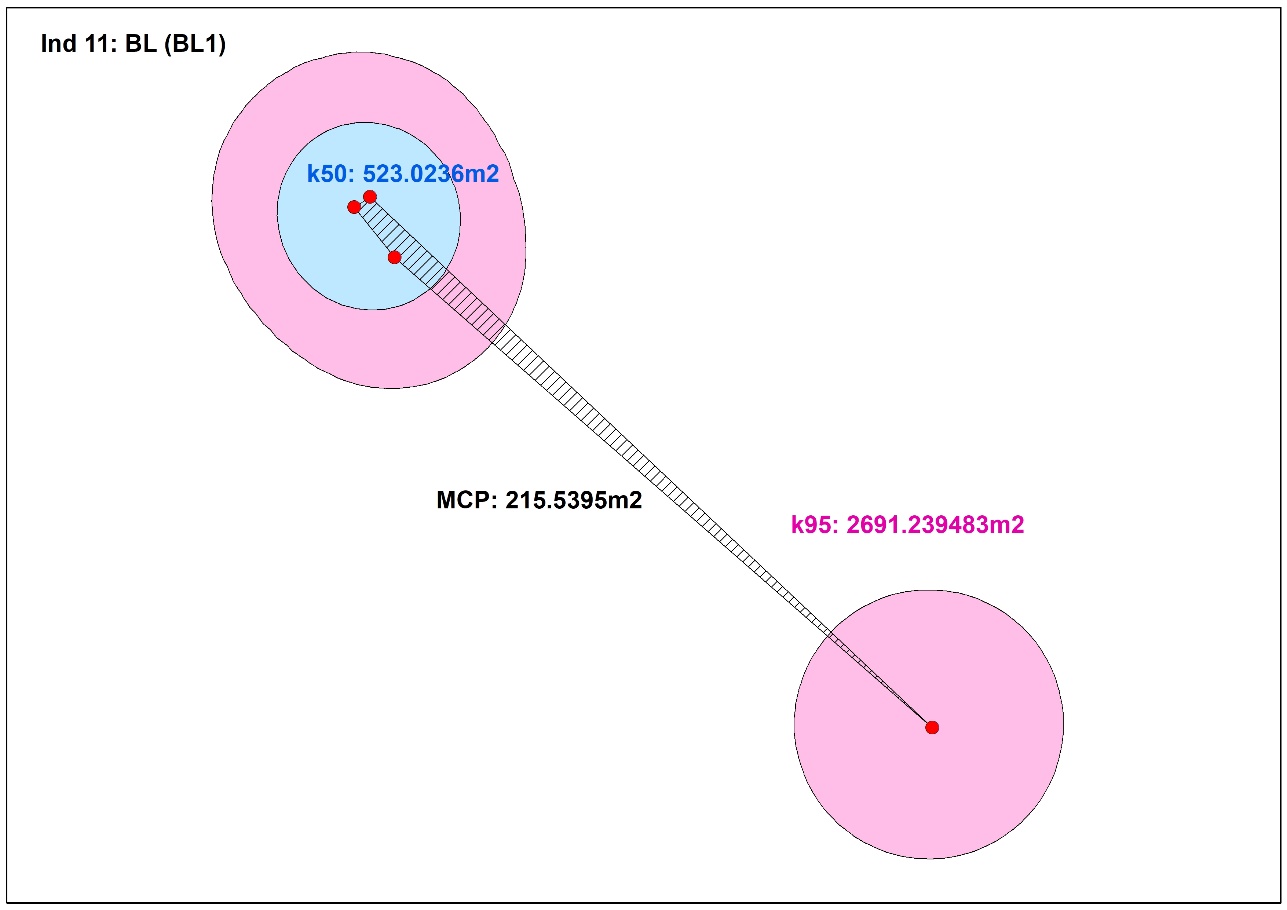


**Figure S1** Example explaining why in some cases the MCPs have a lower value than K50 and K95. This usually occurs when calculations are generated from a sample with few points.
